# Total IgE levels in patients with hematologic malignancies^☆^

**DOI:** 10.1016/j.waojou.2025.101050

**Published:** 2025-04-22

**Authors:** Parisa Amjadi, Fatemehsadat Hosseini, Ehsan Zaboli, Mohammad Eslami-Jouybari, Hossein Asgarian-Omran, Akbar Hedayatizadeh-Omran, Versa Omrani-Nava, Reza Alizadeh-Navaei

**Affiliations:** a Gastrointestinal Cancer Research Center, Non-communicable Diseases Institute, Mazandaran University of Medical Sciences, Sari, Iran; b Department of Immunology, School of Medicine, Mazandaran University of Medical Sciences, Sari, Iran

**Keywords:** Total IgE, Hematologic malignancies, Allergy

## Abstract

**Background:**

Immune system compartments have a crucial role in the progression or suppression of cancer. Hematologic malignancies are among the most common cancers, and there is conflicting research regarding the role of their relationship with allergies and IgE levels. This study aimed to compare total IgE levels in patients with hematologic malignancies to those in non-cancer controls.

**Methods:**

In this cross-sectional study in 2023, sixty patients with hematological malignancies and 90 non-cancer patients attending Sari Imam Hospital were evaluated for total IgE levels along with demographic data. The convenience sampling method was used for the selection of both patients and controls. IgE levels were measured in both groups ELISA method. The analysis was performed using SPSS 20.

**Results:**

The most common types of malignancy were ALL (23.3%) and multiple myeloma (23.3%). The median IgE level in patients (7.08, IQR:2.07–19.07) was significantly lower than the control group (42.41, IQR:11.99–145.57). The odds ratio for hematological malignancy associated with low total IgE was 6.79 (CI 95%: 2.90–15.92). After adjusting for age and sex, the adjusted odds ratio increased to 15.89 (CI 95%: 4.14–60.95).

**Conclusion:**

Patients with hematological cancers have significantly lower IgE levels compared with individuals with no cancer.

## Introduction

Hematologic malignancies are among the most common cancers, and based on the Global Burden of Disease study, incident cases of hematologic malignancies have been increasing over the past 30 years.^1^ Hematologic malignancies, similar to other cancers, develop from a combination of genetic and environmental factors.^2^ Allergy is a hypersensitivity reaction initiated by specific immunological mechanisms.^3^ The available evidence shows that there are 2 opposing views regarding the relationship between allergies and cancer. At one point, it was assumed that allergies might reduce the risk of cancer. In this hypothesis, increased immune surveillance following excessive immune responses may prevent the occurrence or progression of cancer due to the production of tumor-specific immunoglobulin E (IgE) and stimulation of the antitumor effects of eosinophils, basophils, and mast cells.^4^^,^^5^ The alternate hypothesis involves a change in T-helper balance. The predominance of TH2 over TH1 underlies allergic sensitization reactions and is thought to divert the immune response away from TH1, which promotes tumor destruction.^6^ In addition, allergic inflammation may lead to the initiation and progression of cancer directly or through indirect mechanisms.^7^ About role IgE in immunotherapy literature show that IgE antibodies, demonstrate high affinity for Fcε receptors on immune cells, facilitating tumoricidal activity and antibody-dependent cellular cytotoxicity, additionally IgE can stimulate immune cells, promoting a pro-inflammatory environment that enhances macrophage infiltration and anti-tumor signaling pathways in the tumor microenvironment^8^

Despite extensive research into the immune dysregulation associated with hematologic malignancies, limited attention has been directed towards the potential alteration of total IgE levels in these patients and there were some controversies about the role of IgE in malignancy, for instance, Ferastraoaru and Rosenstreich found that IgE-deficient patients have higher rates of newly diagnosed malignancies compared to non–IgE-deficient individuals.^9^ In contrast, Melbye et al reported no association between lymphoma and specific IgE reactivity.^10^ Additionally, another study indicated that total IgE levels were higher in patients with primary cutaneous B cell lymphoma compared to a control group.^11^ If hematologic malignancies are found to alter IgE levels, this could lead to the development of IgE-based biomarkers for early cancer detection and risk stratification. So, this study aimed to determine total IgE levels in patients with hematologic malignancies in comparison with non-cancer patients.

## Methods

This cross-sectional study was conducted on patients suffering from various hematological malignancies who were referred to the chemotherapy center of Sari Imam Hospital before starting chemotherapy in 2023. The control group was non-cancer people that was admitted for surgical and internal medicine reason at this hospital that was selected by call at same time related to case selection. The convenience sampling method was used for the selection of both patients and controls. The sampling in both the case and control groups was convenient. Based on significance level of 0.05, 90% power, expected proportion of 0.33 in 1 group and 0.08 in the other (based on the study of Whiteside et al^12^) using STATA software, the sample size was calculated as 60 per group, though ultimately 60 cases and 90 controls were included. The diagnosis of blood malignancies was established based on clinician diagnosis, histology and flowcytometry. The inclusion criteria in the case group were patients with newly diagnosed hematological malignancies and before first cycle of chemotherapy with an age older than 16 years, and in the control group the age of more than 16 years. The exclusion criteria in the case and control group were patients with autoimmune diseases and immunodeficiency and chronic hepatitis B and C infections, as well as AIDS and smoking. The IgE serum concentration was determined by the ELISA method using the Total IgE ELISA kit (PISHTAZTEB DIAGNOSTICS, Iran). Briefly, 20 μL of serum were dispensed in appropriate wells. After 30 min incubation, the wells were washed and Anti-IgE-HRP conjugate was added for a further 30 min. After washing, the chromogen/substrate solution was added for 15 min and the optical density (OD) was measured at 450/630 nM. The results were expressed as units per milliliter according to a calibrator curve. Based on manual of kit, a value ≥ 40 U/ml was considered as normal. The total IgE level, along with age, gender, and type of cancer, were recorded for the subjects. The analysis was performed using SPSS 20. Mann-Whitney was used to compare IgE concentration between patients and controls and different type of malignancy as Post-hoc test after Kruskal-Wallis, T-test for age differences between groups, Kruskal-Wallis for IgE levels among different types of hematologic malignancies, Fisher's exact test for sex distribution and IgE level categories (low vs normal) between groups and Chi-square for age groups between patients and controls. The histogram drawing method was used to check the normality of the distribution of quantitative variables. The crude Odds Ratio effect size with a 95% confidence interval was calculated using logistic regression and adjusted Odds Ratio was reported to control of age and gender variables as confounders.

## Results

**Fig. 1:**
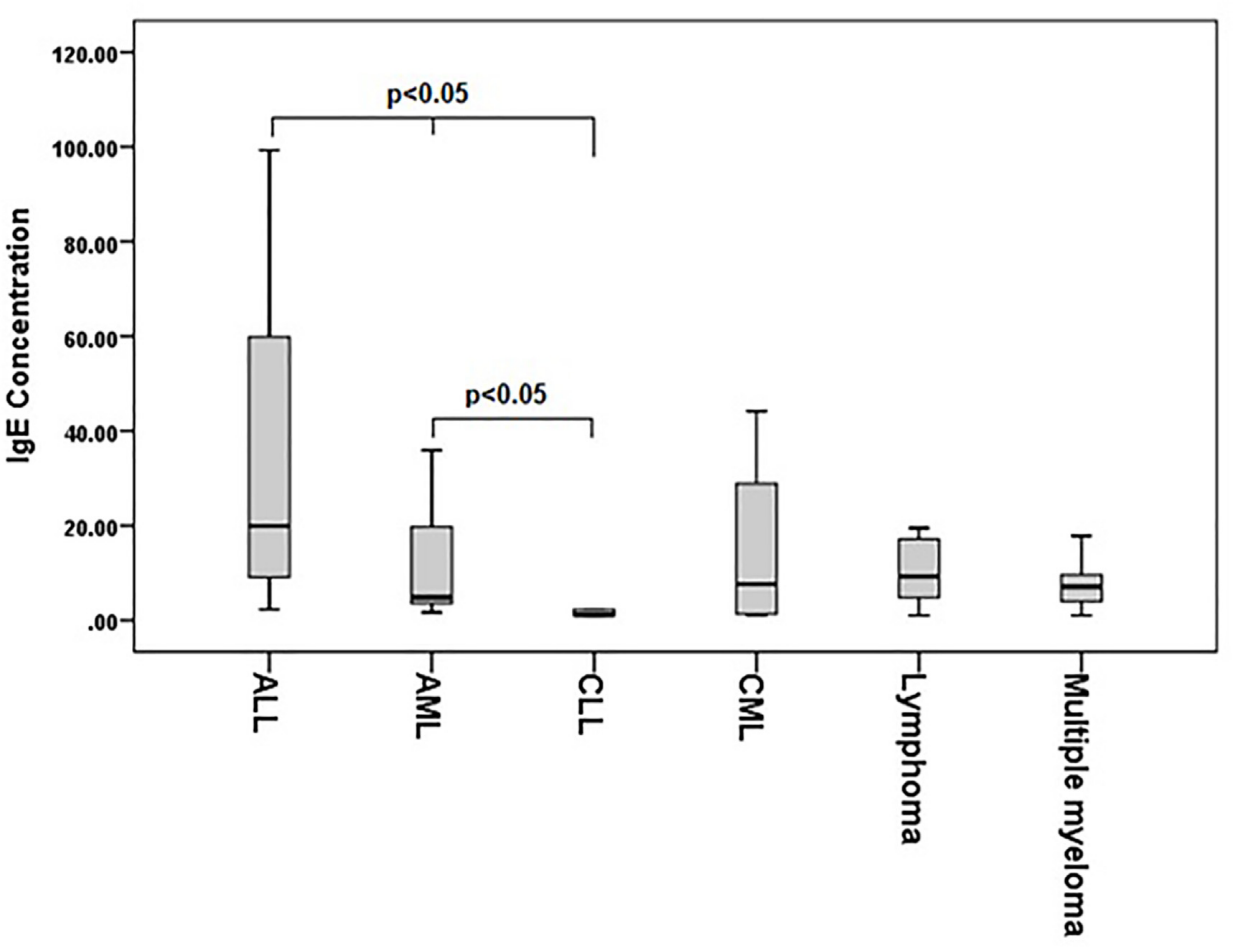
Total IgE levels (IU/ml) in different malignancy types.

The present study was conducted on 60 patients with hematologic malignancies and 90 healthy individuals without cancer as a control group. There were significant differences in the mean age (p = 0.029) and gender distribution (p = 0.000) between patients and controls. The most common type of malignancy was ALL (23.3%) and Multiple myeloma (23.3%). The median IgE level in patients was significantly lower than in the control group (p = 0.000). Among case patients, 52 (86.7%) had IgE levels below the normal range, compared to 51.5% in the control group, and this difference (p = 0.000) was statistically significant (Table 1). T Subjects with low IgE levels had a significantly higher odds of hematological malignancy compared to those with normal IgE levels (OR = 6.79). According to the significant difference of age and gender between the 2 groups, the odds ratio was adjusted for these factors, resulting in an adjusted OR of 15.89 (Table 2). Comparing the amount of total IgE between different malignancies, there was a significant difference (P = 0.002), and Post-hoc test show that the significant difference between ALL and AML (P = 0.042) and CLL (P = 0.000), as well as AML and CLL (P = 0.005) (Fig. 1).

**Table 1:** Subject characteristics in hematological malignancy and control group.

Variable	Hematological malignancy	Control	P-value
Age; year (mean ± SD)	53.08 ± 17.576	46.94 ± 16.10	0.029
Age group N(%)			
<40 year	17 (28.3)	29 (32.2)	0.011
41–60 year	22 (36.7)	48 (53.3)	
≥61 year	21 (35)	13 (14.4)	
Female; N(%)	15 (25)	79 (87.8)	0.000
Male; N(%)	45 (75)	11 (12.2)	
Type of malignancy N(%)			
All	14 (23.3)		
AML	10 (16.7)		
CLL	11 (18.3)		
CML	4 (6.7)		
Lymphoma	7 (11.7)		
Multiple myeloma	14 (23.3)		
IgE level; IU/ml median (Q1-Q3)	7.08 (2.07–19.07)	42.41 (11.99–145.57)	0.000
Low level (≤40 IU/ml)	52 (86.7)	44 (48.9)	0.000
Normal (>40 IU/ml)	8 (13.3)	46 (51.1)

**Table 2:** Odds ratios of hematological malignancies associated with Low Levels of IgE: Crude and adjusted analyses.

	B	S.E	Odds Ratio (CI 95%)	P-value
Crude	1.91	0.43	6.79 (2.90–15.92)	0.000
Adjusted for sex	2.95	0.7	19.13 (4.81–75.62)	0.000
Adjusted for age	2.2	0.47	9.09 (3.61–22.85)	0.000
Adjusted for sex and age∗	2.76	0.68	15.89 (4.14–60.95)	0.000

## Discussion

The present study was conducted to compare the level of total IgE in patients with hematologic malignancies compared to the control group, and the results of the study showed that the level of total IgE in patients with blood cancers was lower than that of the non-malignant group. In a Ferastraoaru et al study in 2020, there was a high chance of malignancy in children with low level IgE compared to children with normal IgE (OR: 7.84, CI95%: 1.94–31.61).^13^ Also, in the study of Ferastraoaru and colleagues in 2018, the investigation of the history of cancer based on different levels of IgE showed a higher prevalence of cancer in people with low IgE compared to people with high IgE (OR: 1.86, CI95%: 1.02–2.4) and very high IgE (OR: 3.07, CI95%:1.03–9.1).^14^ However, in contrast to the result of present and other studies, in Vonderheid et al.'s study in 2019, total IgE levels were higher in patients with primary cutaneous B cell lymphoma compared to a control group.^11^ A higher chance of hematologic malignancies in female patients with a positive history of allergies to plants or grass was observed in the vitamins and lifestyle cohort study.^15^ In contrast, Wulaningsih et al. study show that high level of IgE was inversely associated with the risk of melanoma and combined female breast and gynecological cancers.^7^ The exact mechanism linking IgE to hematological malignancies is complex and not fully understood, but several hypotheses have been proposed to explain the relationship between IgE and cancer including: (a) the chronic inflammation hypothesis, (b) the immune surveillance hypothesis, (c) the prophylaxis hypothesis and (d) the Th2 skewing hypothesis.^16^ One of limitations of this study is the lack of representation of other cancers, such as solid tumors. Additionally, the differences in age and gender distribution between cases and controls, resulting from the study's design not being matched, That we addressed this concern in our analysis. As future research directions, this is a cross-sectional study and we need larger and prospective studies in this concept.

## Conclusion

The results of the present study showed that the level of IgE decreased in patients with hematological malignancies and the calculated odds ratio for this factor was very high, which can indicate the importance of the issue for development of IgE-based biomarkers for early cancer detection.

## Abbreviations

IgE, Immunoglobulin E; ALL, Acute lymphocytic leukemia; AML, Acute myeloid leukemia; CLL, Chronic lymphocytic leukemia; IQR, Interquartile range; CI, Confidence interval; OR, Odds ratio; AIDS, Acquired immunodeficiency syndrome; OD, Optical density.

## Availability of data and materials

The datasets analyzed during the current study are available from the corresponding author on reasonable request.

## Author contributions

RAN contributed to the study conception and design. PA performed the experiments. RAN analyzed the data and wrote the first draft. All authors reviewed the manuscript draft and revised it critically. All authors read and approved the final manuscript.

## Ethics approval

This study was conducted with the code of ethics IR.MAZUMS.IMAMHOSPITAL.REC.1400.018 and was performed in accordance with the Declaration of Helsinki.

## Consent for publication

All authors have consented to the publication of the current manuscript content.

## Funding

This work was supported by 10.13039/501100004160Mazandaran University of Medical Sciences.

## Declaration of competing interest

The authors have no relevant financial or non-financial interests to disclose.
